# Residual gastritis associated with *Strongyloides stercoralis* infection: A case report

**DOI:** 10.1097/MD.0000000000039714

**Published:** 2024-09-27

**Authors:** Shanshan Cai, Miaomiao Zhou, Yulian Zhang, Wei Luo, Bushan Xie

**Affiliations:** a Department of Gastroenterology, Jiangxi Provincial Key Laboratory of Digestive Diseases, Jiangxi Clinical Research Center for Gastroenterology, Digestive Disease Hospital, The First Affiliated Hospital, Jiangxi Medical College, Nanchang University, Nanchang, Jiangxi, China.

**Keywords:** case report, residual gastritis, stercoralis infection, Strongyloides, vomit

## Abstract

**Rationale:**

*Strongyloides stercoralis*, a rare human intestinal parasite, poses a significant health risk, capable of causing lifelong infection and even mortality due to its atypical manifestation of symptoms. In this case report, we reported a case of a patient diagnosed with *S. stercoralis* infection of the residual stomach and meticulously detail its treatment process, offering valuable insights and a reference point for clinicians.

**Patient concerns:**

we report a case of infection caused by *S. stercoralis* after subtotal gastrectomy (Billroth type II) in a 47-year-old middle-aged man. It presents with recurrent nausea and vomiting, accompanied by intermittent food residue vomiting and constipation.

**Diagnoses:**

Upon endoscopic examination, we observed mucosal swelling and erosion in the anastomosis and output ring of stomach, while pathological analysis confirmed the presence of *Strongyloides stercoralis* eggs. Subsequently, the administration of albendazole for anti-infection treatment proved to be effective, thereby reinforcing the diagnosis of *S. stercoralis* infection.

**Intervensions:**

The patient underwent aggressive management including fasting, fluid replacement, anti-infection therapy, albumin supplementation, and albendazole treatment at a dose of 300 mg/kg/day for 3 days to eliminate the parasite

**Outcomes:**

After treatment, the patient’s symptoms of nausea, vomiting, and constipation were alleviated and returned to normal upon discharge. Over the subsequent 3 years, the patient reported no instances of vomiting and experienced a recovery of digestive function compared to their discharge status.

**Lessons:**

*S. stercoralis* infection is relatively rare in the remnant stomach, endoscopic and pathological examination may be one of the important methods to diagnose *S. stercoralis* infection, and it is effective to treat albendazole according to the course of treatment.

## 1. Introduction

*S. stercoralis* infections are usually chronic and long-term. Altered immune status increases the risk of hyperinfection syndrome, transmission, etc.^[1]^ *S. stercoralis* infection is mainly presented with gastrointestinal and pulmonary involvement and is endemic in tropical and subtropical regions of the world such as Europe and Italy.^[2]^ This case reports a patient who was admitted to the Department of Gastroenterology of the First Affiliated Hospital of Nanchang University. The patient was diagnosed with *S. stercoralis* infection of the stomach. In this case study, we reviewed the diagnosis and treatment of the patient. Therefore, more patients with *S. stercoralis* infection can be diagnosed and treated promptly to avoid misdiagnosis.

## 2. Case report

A 47-year-old middle-aged male presented with recurrent nausea and vomiting since undergoing a subtotal gastrectomy (Billroth II type) 20 years ago, accompanied by intermittent vomiting of food residues. External consultations suggested residual gastritis. Over the past 5 days, the nausea and vomiting have intensified, with increased volume of vomitus, and bowel movements requiring assistance with glycerin suppositories. The patient has lost 6.5 kg in recent weight. Past health status was average, with a history of surgery, specifically a subtotal gastrectomy (Billroth II type) 20 years ago, penicillin allergy, a 20-year history of alcohol consumption, and no special medical history such as contact with epidemic water or epidemic areas. On admission, physical examination revealed no specific signs in the heart and lungs. The abdomen was flat and soft, with a visible longitudinal surgical scar of approximately 20 cm in length in the mid-abdomen. There were no positive signs such as tenderness or rebound tenderness.

**Figure 1.**
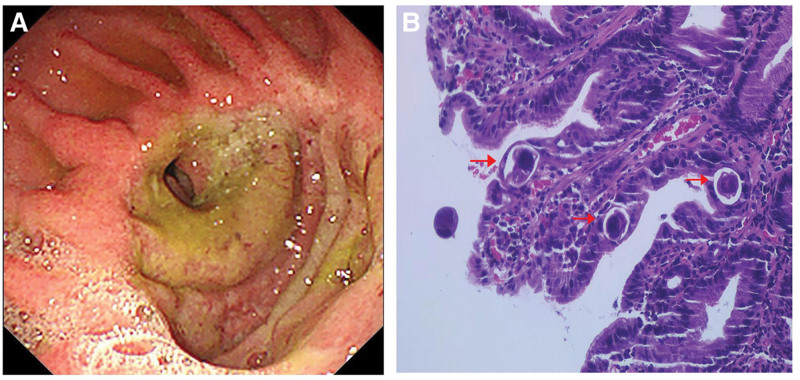
Gastroscopy showed swelling and erosion of the mucosa at the anastomosis and output loop, with purulent moss on the surface (A). Many eggs in different stages of maturation are located in the crypts. Eggs in the crypt have developed larvae within a thin egg shell (B).

Relevant laboratory investigations conducted upon admission showed an elevated white blood cell count (WBC) of 16.64×10^9^/L (normal range: 3.5–9.5 × 10^9^/L), eosinophil count of 1.51 × 10^9^/L (normal range: 0.02–0.52 × 10^9^/L), and a C-reactive protein (CRP) concentration of 17.30 mg/L (normal range: 0–8 mg/L), indicating elevated inflammatory markers. Abdominal CT suggested postoperative changes in the stomach, with no significant thickening of the anastomotic wall, and multiple enlarged lymph nodes observed at the root of the jejunal mesentery. During gastroscopy, mucosal swelling and erosion were observed at the anastomoses and output loop (Fig. 1A), and *S. stercoralis* infection was observed on pathological examination (Fig. 1B).

After multi-disciplinary consultations to rule out vomiting caused by other factors, it was recommended to proceed with antiparasitic treatment. The patient underwent active treatment, which included fasting during hospitalization, along with rehydration and nutrition therapy consisting of electrolytes, fat milk, glucose, and albumin supplementation. Additionally, anti-infection treatment with latoxefe and albendazole (dosage of 300 mg/kg/day for 3 days) was administered to eliminate parasites. Subsequently, the patient’s symptoms of nausea, vomiting, and constipation were alleviated and returned to normal upon discharge.

## 3. Follow-up record

Over a 3-year period of telephonic follow-up, we have systematically evaluated the patient’s health status. Clinical data indicates that while the patient has not undergone a repeat gastroscopy since discharge, they have reported a reduction in vomiting symptoms compared to their discharge status, suggesting a degree of recovery in digestive function. However, the patient experiences occasional episodes of acid reflux, a symptom potentially attributed to anatomical alterations in the stomach following previous subtotal gastrectomy, which in turn affects normal gastric acid secretion and emptying mechanisms.

Currently, the patient is on continuous medication, specifically oral Albendazole at a dosage of 300 mg/kg/day, administered in a periodic regimen of twice a year. Although Albendazole is primarily a broad-spectrum antiparasitic agent and does not directly target acid reflux symptoms, its potential gastrointestinal regulatory effects or supportive role in a combined treatment plan may contribute to maintaining the patient’s overall health condition.

It is noteworthy that given the patient’s history of subtotal gastrectomy and occasional acid reflux, it is recommended to intensify monitoring of their gastrointestinal symptoms during future follow-ups and consider timely arrangements for repeat gastroscopy. This will enable the assessment of gastric structural recovery, the risk of potential complications such as anastomotic stenosis and residual gastric cancer, and the efficacy of the current medication regimen. Additionally, patients should be counseled on maintaining a healthy diet, avoiding stimulating foods, and undergoing regular follow-ups for treatment adjustments to ensure comprehensive and professional medical care.

## 4. Discussion and conclusions

*S. stercoralis* were first studied in 1876^[3]^ and often parasitize in the human intestinal cavity,^[4]^ and relatively few reports have involved the stomach.^[5–7]^ Although the stomach is not an ideal parasitic site for *S. stercoralis*, reduced gastric acid secretion may favor infection and invasion of the stomach.^[8]^ According to the literature, it is very rare for *S. stercoralis* to be carried in the remnant stomach after Billroth’s II resection.

The full life cycle, pathology, and clinical features of human infection were intensively studied in the 1930s^3^. After developing twice, the larva transformed into an infectious third stage larva (L3) that was capable of infecting a new host by penetrating intact skin. Larvae thrived in warm and moist soil.^[9,10]^ The threadworm *S. stercoralis* is a soil-transmitted parasite, and most infected patients contract the disease because of exposure to contaminated soil in epidemic areas like the patient in this case.^[11,12]^ The patient was a farmer who worked on a farm and was therefore exposed to contaminated soil.

*S. stercoralis* infection is one of the most neglected diseases and can lead to lifelong infection and mortality because of atypical symptoms.^[13]^ *S. stercoralis* in endemic areas can persist for decades in the larval self-infection cycle, with some studies reporting infections lasting longer than 75 years.^[14]^ Misdiagnosis, inadequate treatment, and immunosuppression due to hyperinfective syndromes are common and lead to high mortality. In this case, *S. stercoralis* may have been present in the patient’s body for decades and remained chronically infected. The patient’s preexisting medical conditions included Billroth’s II subtotal gastrectomy and the gastroscopic report showing anastomotic inflammation may indicate the combination of *S. stercoralis* infection and anastomotic inflammation contributed to the patient’s chronic vomiting and other symptoms.

Studies have shown there is a high risk of *S. stercoralis* infection among HIV/AIDS patients, HTLV-1 patients, alcoholics, diarrhea patients, malignant tumor patients, immunocompromised patients, and children.^[3]^ Due to the patient’s history of drinking alcohol, protective barriers may be destroyed, resulting in an increased risk of exposure to *S. stercoralis*.^[15–17]^ In addition, the patient underwent Billroth’s II resection for gastric carcinoma, which resulted in a reduction of gastric acid and destruction of barriers. All of these factors made the gastric invasion of *S. stercoralis* possible.

When evaluating suspected *S. stercoralis* infection, attention should be paid to the following: main symptoms: Skin manifestations: perianal creeping eruption is rare, while pruritus (itching) in the feet is common; Respiratory system: wheezing, coughing, and low-grade fever, indicative of larval migration to the lungs; Abdominal/gastrointestinal tract: epigastric tenderness, intermittent diarrhea, nausea, and vomiting. In severe cases, abdominal pain intensifies, accompanied by frequent vomiting, persistent diarrhea, and potentially intestinal obstruction or sepsis; Recurrent skin symptoms: pruritus and recurrent skin rashes (e.g., chronic urticaria), reflecting the host’s immune response.^[18–20]^ Key points of gastric *S. stercoralis* infection: prominent gastrointestinal symptoms, particularly aggravated epigastric pain, vomiting, and diarrhea. Differential diagnosis: Parasitic infections: such as hookworm and ascariasis, requiring parasitological examination for differentiation; Non-infectious gastrointestinal diseases: including inflammatory bowel disease and functional gastrointestinal disorders, which require comprehensive evaluation for exclusion; Respiratory diseases: such as asthma and COPD, where symptoms must be distinguished from those caused by *S. stercoralis* infection; Neurological diseases: notably viral and tuberculous meningitis. In cases of meningitis, cerebrospinal fluid examination and etiological testing are necessary.

The symptoms of *S. stercoralis* infection are complex and require a comprehensive assessment of symptoms, signs, and diagnostic tests for definitive diagnosis.

Diagnosis and effective treatment are essential to eradicate the infection. The standard diagnosis relies on the presence of parasites in the feces. However, 7 fecal tests are required to achieve 100% sensitivity due to the low parasite load. These limitations make fecal microscopes a rather controversial gold standard.^[3,4]^ Thus, early endoscopic diagnosis and pathological examination of strongyloidiasis can have a marked impact on disease outcome,^[21]^ there have been very few reports regarding the histopathologic yield by endoscopy for strongyloidiasis. In a study by Thompson et al^[22]^ a minimum of 6 biopsies were obtained from each lesion, resulting in a 100% histopathologic yield from the 6 patients. The fecal tests conducted on the patient in this case did not reveal *S. stercoralis*, but the pathological examination confirmed *S. stercoralis* infection in the stomach of the patient. Thus endoscopic observation and biopsies, in addition to gastroduodenal drainage analysis, are important tools for diagnosing strongyloidiasis.

The goals of treatment for *S. stercoralis* infection are as follows: complete elimination of the pathogen; treatment of symptomatic infections; and prevention of complications associated with asymptomatic infection.^[18,23–25]^ Ivermectin (200 μg/day for 1–2 days) is the drug of choice for uncomplicated *S. stercoralis* infection, It is generally considered as an alternative therapy to administer Albendazole 400 mg twice a day for 3–7 days. Ivermectin has been shown to be more curative than Albendazole.^[1,23,24]^ Studies show that treatment with Ivermectin and Albendazole can cure similar numbers of people with *S. stercoralis* infection, but Ivermectin may be better tolerated.^[19,23]^ In this case, the treatment with Albendazole (300 mg/kg/day for 3 days) greatly alleviated the patient’s symptoms, which may confirm that Albendazole is also an effective treatment.

In summary, it is difficult to diagnose *S. stercoralis* infection using routine laboratory and fecal examinations. An accurate diagnosis is made based on a thorough examination of the patient’s history, including their living environment, their lifestyle, their employment, their age, and their personal history, as well as abnormal laboratory tests such as eosinophils, blood sedimentation, white blood cells, and other inflammatory indicators. There was intermittent vomiting in this patient while the initial complete blood count with increasing eosinophilia indicated that the patient had been infected with a parasite, as well as abnormal laboratory tests such as eosinophils, blood sedimentation, white blood cells, and other inflammatory indicators. In this case, the patient presented with intermittent vomiting while the initial complete blood count with increasing eosinophilia indicated the infection of the parasite. Finally, a gastroscopic pathological examination confirmed the diagnosis of *S. stercoralis* infection, which was followed by standardized treatment and discharge after the resolution of symptoms.

There are several limitations to this study. While the diagnosis, treatment process, and outcomes of this case were recorded in detail, the study is restricted to only 1 instance of *S. stercoralis* infection in the stomach due to the rarity of the disease. The small sample size hinders the ability to comprehensively represent the overall characteristics, epidemiological features, and the universality of treatment effects for this condition. Consequently, the findings of this study have limited reference value, necessitating the accumulation of more cases and further research to validate and enhance understanding.

## Acknowledgments

This work was supported by the Jiangxi Provincial Science Foundation (No. 20202ACBL216014).

## Author contributions

**Data curation:** Shanshan Cai, Miaomiao Zhou.

**Formal analysis:** Miaomiao Zhou.

**Project administration:** Wei Luo.

**Software:** Wei Luo, Bushan Xie.

**Supervision:** Bushan Xie.

**Validation:** Yulian Zhang, Bushan Xie.

**Visualization:** Yulian Zhang.

**Writing – original draft:** Shanshan Cai.

**Writing – review & editing:** Shanshan Cai.

## References

[1] Nutman TB. Human infection with Strongyloides stercoralis and other related Strongyloides species. Parasitology. 2017;144:263–73. 27181117 10.1017/S0031182016000834 PMC5563389

[2] Pane S Sacco A Iorio A Romani L Putignani L. Strongyloides stercoralis Infestation in a Child: how a nematode can affect gut microbiota. Int J Mol Sci. 2021;22:2131. 33669932 10.3390/ijms22042131 PMC7924877

[3] Schar F Trostdorf U Giardina F. Strongyloides stercoralis: global Distribution and Risk Factors. PLoS NeglTrop Dis. 2013;7:e2288. 10.1371/journal.pntd.0002288 PMC3708837 23875033

[4] Teixeira MC Pacheco FT Souza JN Silva ML Inês EJ Soares NM. 2016. Strongyloides stercoralis infection in alcoholic patients. Biomed Res Int. 2016;4872473. 10.1155/2016/4872473 PMC5220430 28105424

[5] Kim J Joo HS Kim DH Lim H Kang YH Kim MS. A case of gastric strongyloidiasis in a Korean patient. Korean J Parasitol. 2003;41:63–7. 12666732 10.3347/kjp.2003.41.1.63 PMC2717484

[6] Patra AA Nath P Pati GK. Strongyloides infection presenting as proximal small intestinal obstruction. ACG Case Reports J. 2019;6:e00124. 10.14309/crj.0000000000000124 PMC6722340 31616778

[7] Yaldiz M Hakverdi S Aslan A Temiz M Culha G. Gastric infection by Strongyloides stercoralis: a case report. Turkish J Gastroenterol. 2009;20:48–51. 19330735

[8] Giannella RA Broitman SA Zamcheck N. Influence of gastric acidity on bacterial and parasitic enteric infections. A perspective. Ann Intern Med. 1973;78:271–6. 4567180 10.7326/0003-4819-78-2-271

[9] Concha R Harrington W Jr. Rogers AI. Intestinal strongyloidiasis: recognition, management, and determinants of outcome. J Clin Gastroenterol. 2005;39:203–11. 15718861 10.1097/01.mcg.0000152779.68900.33

[10] Genta RM. Dysregulation of strongyloidiasis: a new hypothesis. Clin Microbiol Rev. 1992;5:345–55. 1423214 10.1128/cmr.5.4.345 PMC358253

[11] Senephansiri P Laummaunwai P Laymanivong S Boonmar T. Status and risk factors of strongyloides stercoralis infection in rural communities of Xayaburi Province, Lao PDR. Korean J Parasitol. 2017;55:569–73. 29103274 10.3347/kjp.2017.55.5.569 PMC5678471

[12] Widjana DP Sutisna P. Prevalence of soil-transmitted helminth infections in the rural population of Bali, Indonesia. Southeast Asian J Trop Med Public Health. 2000;31:454–9. 11289000

[13] Arifin N Hanafiah KM Ahmad H Noordin R. Serodiagnosis and early detection of Strongyloides stercoralis infection. J Microbiol Immunol Infect. 2019;52:371–8. 30482708 10.1016/j.jmii.2018.10.001

[14] Scaramozzino N Crance JM Jouan A DeBriel DA Stoll F Garin D. Comparison of flavivirus universal primer pairs and development of a rapid, highly sensitive heminested reverse transcription-PCR assay for detection of flaviviruses targeted to a conserved region of the NS5 gene sequences. J Clin Microbiol. 2001;39:1922–7. 11326014 10.1128/JCM.39.5.1922-1927.2001 PMC88049

[15] Pavia CS La Mothe M Kavanagh M. Influence of alcohol on antimicrobial immunity. Biomed Pharmacother = Biomedecine & pharmacotherapie. 2004;58:84–9. 14992788 10.1016/j.biopha.2003.12.005

[16] Szabo G. Consequences of alcohol consumption on host defence. Alcohol alcoholism (Oxford, Oxfordshire). 1999;34:830–41. 10.1093/alcalc/34.6.830 10659718

[17] Szabo G Mandrekar P. A recent perspective on alcohol, immunity, and host defense. Alcohol Clin Exp Res. 2009;33:220–32. 19053973 10.1111/j.1530-0277.2008.00842.x PMC3787826

[18] Keiser PB Nutman TB. Strongyloides stercoralis in the Immunocompromised Population. Clin Microbiol Rev. 2004;17:208–17. 14726461 10.1128/CMR.17.1.208-217.2004 PMC321465

[19] Krolewiecki A Nutman TB. Strongyloidiasis: a neglected tropical disease. Infect Dis Clin North Am. 2019;33:135–51. 30712758 10.1016/j.idc.2018.10.006 PMC6367705

[20] Marcos LA Terashima A Canales M Gotuzzo E. Update on strongyloidiasis in the immunocompromised host. Curr Infect Dis Reports. 2011;13:35–46. 10.1007/s11908-010-0150-z 21308453

[21] Kishimoto K Hokama A Hirata T. Endoscopic and histopathological study on the duodenum of Strongyloides stercoralis hyperinfection. World J Gastroenterol. 2008;14:1768–73. 18350608 10.3748/wjg.14.1768 PMC2695917

[22] Thompson BF Fry LC Wells CD. The spectrum of GI strongyloidiasis: an endoscopic-pathologic study. Gastrointest Endosc. 2004;59:906–10. 15173813 10.1016/s0016-5107(04)00337-2

[23] Henriquez-Camacho C Gotuzzo E Echevarria J White AC Jr. Terashima A. Ivermectin versus albendazole or thiabendazole for Strongyloides stercoralis infection. Cochrane Database Syst Rev. 2016;2016:Cd007745. 26778150 10.1002/14651858.CD007745.pub3 PMC4916931

[24] Suputtamongkol Y Premasathian N Bhumimuang K. Efficacy and safety of single and double doses of ivermectin versus 7-day high dose albendazole for chronic strongyloidiasis. PLoS NeglTrop Dis. 2011;5:e1044. 10.1371/journal.pntd.0001044 PMC3091835 21572981

[25] Toledo R Muñoz-Antoli C Esteban JG. Strongyloidiasis with emphasis on human infections and its different clinical forms. Adv Parasitol. 2015;88:165–241. 25911368 10.1016/bs.apar.2015.02.005

